# Roux-en-O after revisional bariatric surgery: a rare complication following OAGB to RYGB conversion

**DOI:** 10.1093/jscr/rjag547

**Published:** 2026-07-07

**Authors:** Mohammad Almayouf, Abdulrahman Alswat, Awadh Alqahtani

**Affiliations:** College of Medicine, Department of Surgery, Prince Sattam bin Abdulaziz University, Alkharj, 11942 Saudi Arabia; College of Medicine, Department of Surgery, King Saud University, Riyadh, Saudi Arabia; College of Medicine, Department of Surgery, King Saud University, Riyadh, Saudi Arabia

**Keywords:** gastric bypass, bariatric surgery, reoperation, postoperative complications, bile reflux, Roux-en-O configuration

## Abstract

Roux-en-O (ROGB) configuration is a rare technical complication caused by incorrect limb orientation, resulting in biliopancreatic secretions draining into the gastric pouch. We report a 47-year-old female with multiple prior bariatric surgeries who underwent conversion to Roux-en-Y gastric bypass for refractory gastroesophageal reflux disease and developed persistent food intolerance, bilious vomiting, and reflux. ROGB was suspected on imaging and confirmed intraoperatively. Surgical revision with reconstruction of the gastrojejunostomy and jejunojejunostomy restored proper limb orientation, leading to complete symptom resolution. This case emphasizes the importance of careful limb identification during revisional bariatric surgery to prevent this rare complication.

## Introduction

Roux-en-Y gastric bypass (RYGB) remains one of the most effective metabolic and bariatric surgical procedures. It is well established as both primary and revisional operation, providing durable weight reduction and significant improvement in obesity-related comorbidities [1, 2]. Achieving optimal perioperative outcomes, however, requires substantial technical experience. Evidence suggests that surgeons must perform ~500 RYGB procedures to attain proficiency and minimize procedure-related complications [3]. Despite its widespread adoption, RYGB requires meticulous attention to operative detail to avoid technical errors. One such error is the inadvertent creation of the ‘Roux-en-O’ configuration, which results from incorrect orientation of the bowel limbs. Reported cases of this incorrect configuration have predominantly occurred following primary RYGB.

In this paper, we present a case of Roux-en-O gastric bypass (ROGB) configuration occurring during conversion from a one-anastomosis gastric bypass (OAGB) to RYGB. We describe the mechanism of occurrence, the operative management, and provide a focused review of the existing literature.

## Case report

**Figure 1:**
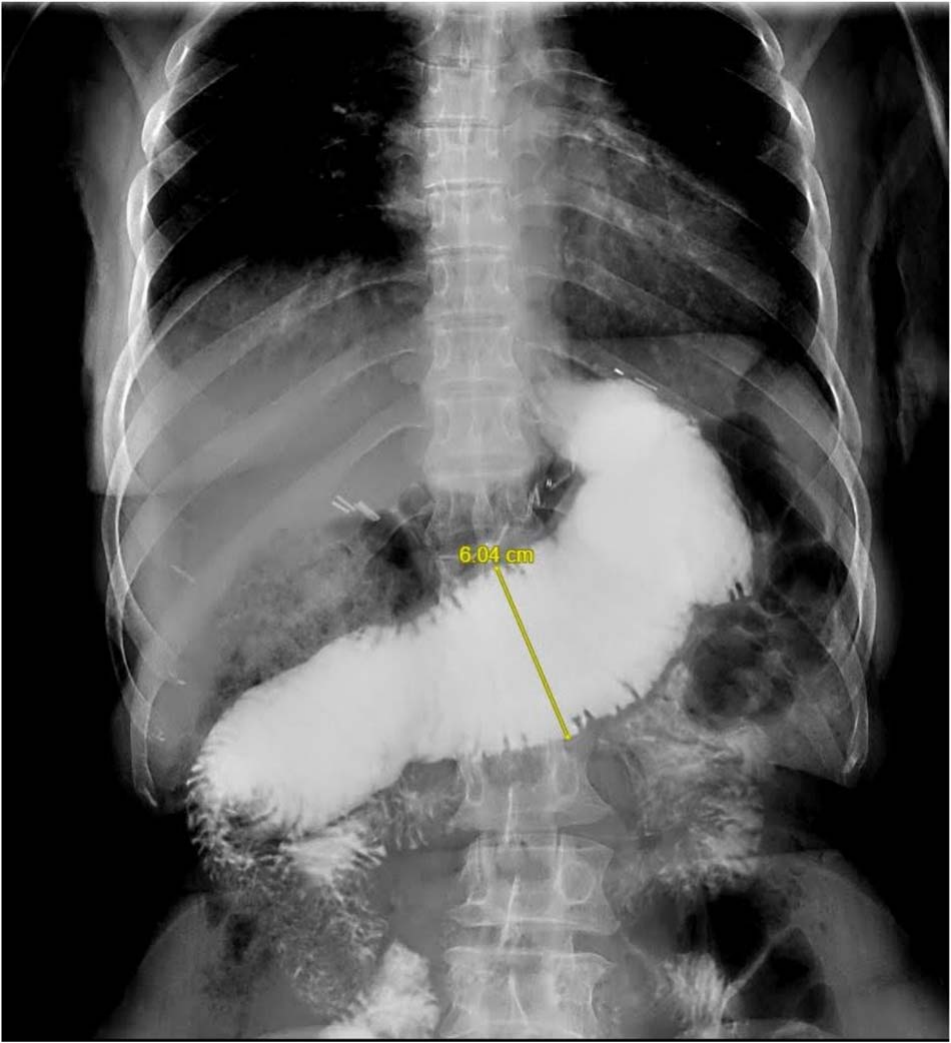
Contrast study showing excessively dilated Roux limb.

A 47-year-old female with longstanding gastro-esophageal reflux disease (GERD) and multiple prior bariatric procedures (LSG 12 years earlier, conversion to OAGB 6 years later, and subsequent conversion to RYGB after iatrogenic perforation during endoscopy) presented with persistent food intolerance, bilious vomiting, and refractory reflux. Upper gastrointestinal (UGI) contrast study showed a dilated alimentary limb with severe reflux, and endoscopy revealed bile in the alimentary limb, raising suspicion of ROGB configuration (Fig. 1). Intraoperative limb tracing confirmed ROGB, with the alimentary limb mesentery continuing toward the ligament of Treitz in a loop configuration. Surgical correction was performed by dismantling the gastrojejunostomy, reconstructing a new gastrojejunostomy, and creating a jejunojejunostomy 100 cm distal to it. Postoperative UGI showed normal contrast flow, and the patient achieved complete symptom resolution with satisfactory weight stabilization.

## Discussion

Incorrect construction of RYGB is rare, and one possible technical error is the ROGB configuration. In this scenario, the bowel limb draining the excluded stomach is inadvertently anastomosed to the gastric pouch, resulting in a closed loop in which biliopancreatic secretions drain into the gastric pouch. This flawed configuration may occur particularly during revisional bariatric procedures, where anatomical alterations and adhesions increase technical complexity. Additionally, obesity itself may increase the technical difficulty of such procedures. Incorrect restoration of gastrointestinal continuity has also been reported in other gastrointestinal surgeries outside bariatric surgery practice. For example, Zorn *et al.* reported two cases of recurrent cholangitis following hepaticojejunostomy [4]. Exploration revealed that the biliary anastomosis had been constructed incorrectly using the wrong limb, resulting in recurrent cholangitis.

The symptoms of ROGB configuration can often be predicted based on the abnormal drainage of biliopancreatic secretions into the gastric pouch. Patients may present with early postoperative bilious vomiting, persistent nausea, abdominal pain, food intolerance, excessive weight loss, and intractable GERD [6, 8]. Diagnosis can be challenging due to the rarity of the condition and requires a high index of suspicion. Reported cases demonstrate significant variability in the time to diagnosis, ranging from identification during the index operation to several months after surgery (Table 1).

**Table 1:** Reports of errant bowel limb construction in gastrointestinal surgeries

Author / Year	Type of surgery	No.	Error type	Presentation	Investigations	Operative findings	Management
Zorn *et al.,* 1999 [4]	RY hepaticojejunostomy	2	Antiperistaltic Roux limb	Recurrent cholangitis	Re-exploration	Reversed limb	Isoperistaltic reconstruction
DiBaise *et al.,*, 2005 [5]	Total gastrectomy, RY-esophagojejunostomy	2	Antiperistaltic Roux limb	Bilious vomiting, aspiration pneumonia, malnutrition	UGI, CT, EGD, manometry	Retrograde phase III contractions confirming a reversed limb	Reorientation of the esophagojejunostomy to an isoperistaltic direction
Nelson *et al.,* 2006 [6]	Open and laparoscopic RYGB	5	Antiperistaltic Roux limb	Bilious vomiting, protein-calorie malnutrition	NA	Antiperistaltic orientation	Reorientation to an isoperistaltic direction
Schrope *et al.,* 2006 [7]	Open ROGB	3	Reverse peristaltic alimentary limb	Obstructive symptoms after surgery	NA	Reverse peristaltic alimentary limb	Not specified
Sherman *et al.,* 2009 [8]	Open and laparoscopic RYGB	4	ROGB	Bilious vomiting, small bowel obstruction.	UGI, CT, small bowel follow-through, EGD	Incorrect JJ, blind limb, excessively long biliopancreatic limb, volvulus	Multiple laparotomies; revision of the JJ and GJ
Sanders *et al.,* 2012 [9]	Laparoscopic RYGB	1	Antiperistaltic secondary to partial obstruction	Nausea, vomiting, abdominal pain, malnutrition	UGI, CT, EGD	Dilated Roux limb with retrograde contractions	Two revisions of the JJ

RY: Roux en Y, RYGB: Roux en Y gastric bypass, ROGB: Roux en O gastric bypass, UGI: upper gastrointestinal series, CT: computed tomography, EGD: esophagogastroduodenoscopy, GJ: gastrojejunostomy, JJ: jejunojejunostomy

UGI contrast studies are commonly used in suspected cases. Radiologic findings suggestive of incorrect configuration include an excessively dilated Roux limb and the observation of peristalsis directed toward the gastric pouch during fluoroscopy [6, 10]. Esophagogastroduodenoscopy can also be helpful, as it may demonstrate bile reflux within the gastric pouch. Another diagnostic modality used to evaluate duodenogastric reflux is technetium-99 m–labeled hydroxyiminodiacetic acid scintigraphy. After intravenous administration, the tracer is excreted by hepatocytes into the bile and can be visualized using gamma cameras. Reflux of the tracer into the gastric pouch suggests abnormal biliary drainage [8]. Some authors have suggested that upper gastrointestinal contrast studies alone may be insufficient to confirm incorrect configuration and advocate for additional diagnostic testing, such as intestinal manometry [11].

Fortunately, technical errors in gastrointestinal reconstruction remain rare. However, with the increasing number of bariatric procedures performed worldwide, revisional bariatric surgery is becoming more common due to various indications, including intractable GERD and bile reflux [12, 13]. To our knowledge, this is the first report describing a ROGB configuration following conversion from OAGB. Two possible mechanisms may explain the occurrence of this configuration.

**Figure 2:**
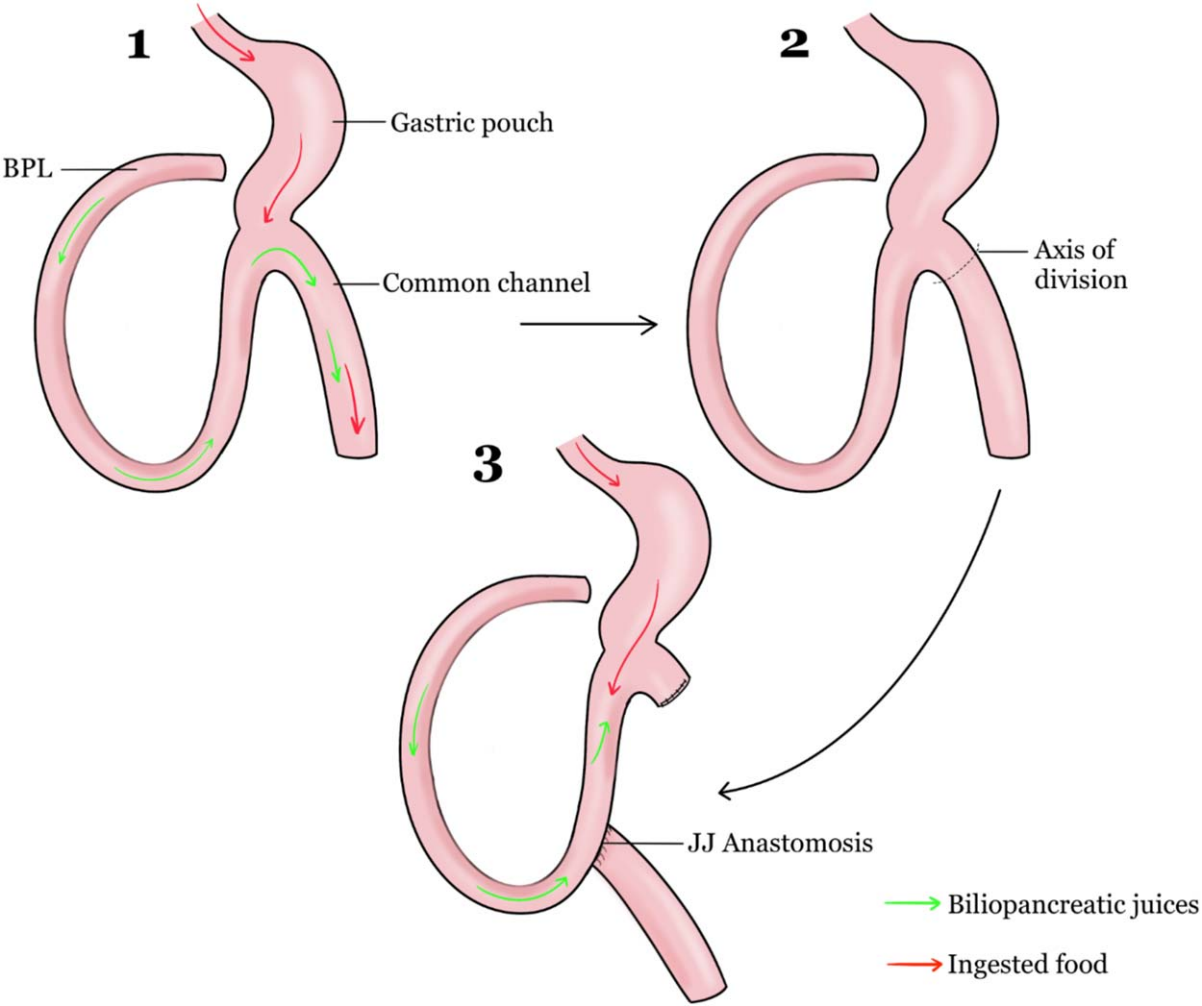
Explanation 1 of mechanism of ROGB construction after Billroth II.

**Figure 3:**
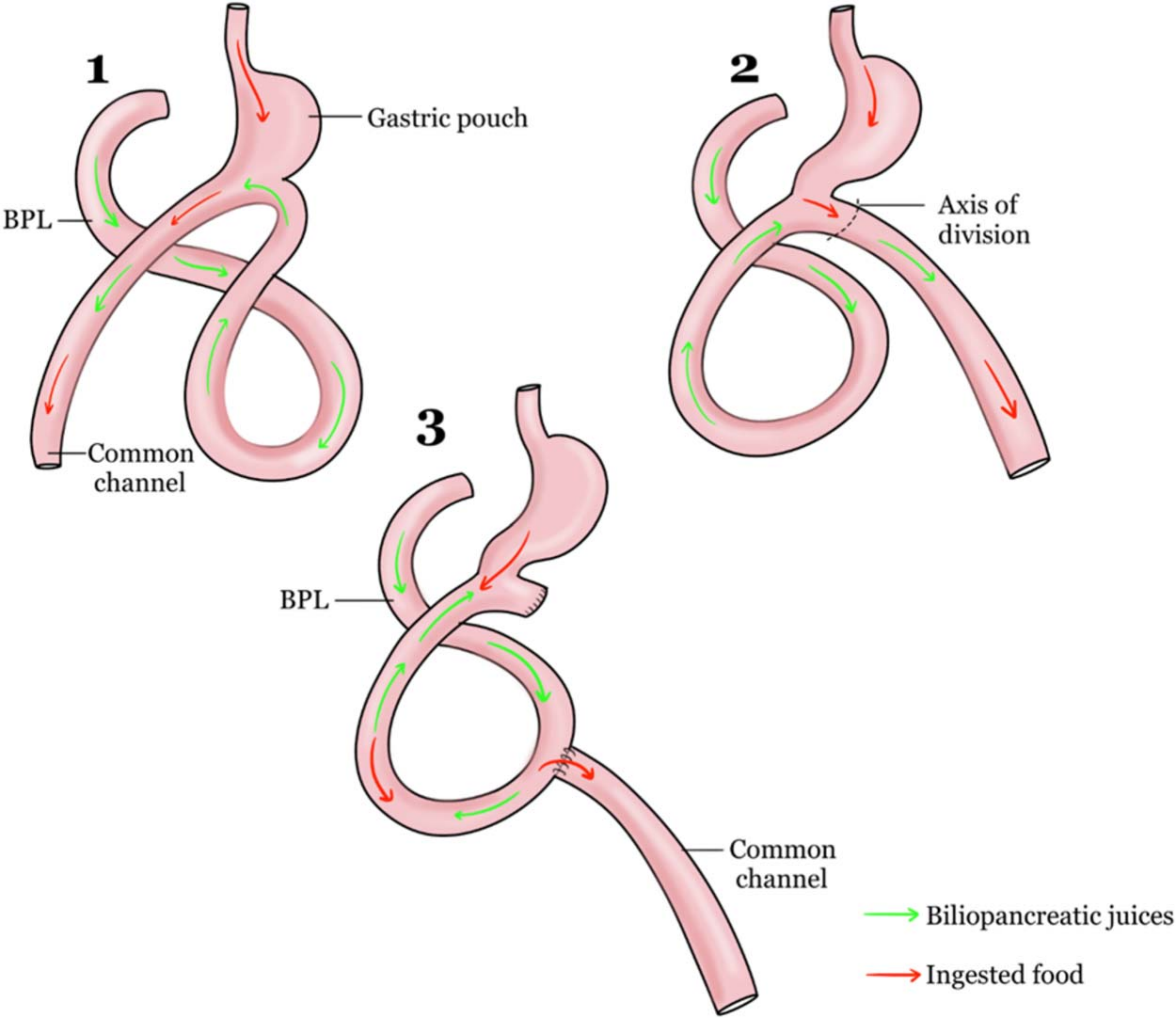
Explanation 2 of mechanism of ROGB construction after OAGB.

The first possibility is that the original OAGB was constructed according to the classic Billroth II, where the biliopancreatic limb is brought from the right side of the patient to the left. During conversion to RYGB, the common channel was been divided and anastomosed to the same biliary limb, resulting in an O-configuration (Fig. 2). The second possibility is that the OAGB was constructed with the biliary limb running from left to right, but the limb became twisted or flipped during conversion. Consequently, the common channel have been inadvertently divided and anastomosed to the biliary limb, again producing the O-configuration (Fig. 3).

During the construction of OAGB, the orientation of the biliary limb to the gastric pouch, i.e. from left to right or right to lift is not stated explicitly in the literature, but the authors who popularized this procedure and large studies are displaying that the biliary limb going from the left to the right side of the patient [14, 15]. We believe that this is the main reason why a ROGB developed in this particular case.

## Conclusion

Roux-en-O configuration is a rare but significant technical complication of RYGB that can be prevented by meticulous surgical technique and careful verification of bowel limb orientation. This case adds to the literature by reporting Roux-en-O following conversion from OAGB to RYGB, highlighting the technical complexity of revisional bariatric surgery and the importance of strict adherence to fundamental reconstructive principles to avoid this preventable complication.

## Author contribution

M.A. contributed to writing of paper, literature review, collecting images. A. Alswat made literature review. A. Alqahtani performed review & editing.

## References

[1] Guimarães M, Osório C, Silva D et al. How sustained is Roux-en-Y gastric bypass long-term efficacy? Obes Surg 2021;31:3623–9. 10.1007/s11695-021-05458-y 34021884 PMC8270797

[2] Uhe I, Douissard J, Podetta M et al. Roux-en-Y gastric bypass, sleeve gastrectomy, or one-anastomosis gastric bypass? A systematic review and meta-analysis of randomized-controlled trials. Obesity 2022;30:614–27. 10.1002/oby.23338 35137548 PMC9303972

[3] Doumouras AG, Saleh F, Anvari S et al. Mastery in bariatric surgery. The long-term surgeon learning curve of Roux-en-Y gastric bypass. Ann Surg 2018;267:489–94. 10.1097/SLA.0000000000002180 28230663

[4] Zorn GL, Wright JK, Pinson CW et al. Antiperistaltic Roux-en-Y biliary-enteric bypass after bile duct injury: a technical error in reconstruction. Sage J 1999;65:581–5. 10.1177/000313489906500614 10366214

[5] DiBaise JK, Iyer K, Thompson JS. Identification and management of an errant antiperistaltic Roux limb after total gastrectomy. Journal of Gastrointestinal Surgery. 2005;9:726–32. 15862271 10.1016/j.gassur.2004.12.004

[6] Nelson LG, Sarr MG, Murr MM. Errant and unrecognized antiperistaltic Roux limb construction during Roux-en-Y gastric bypass for clinically significant obesity. Surg Obes Relat Dis 2006;2:523–7. 10.1016/j.soard.2006.07.009 17015205

[7] Schrope BA, Daud A, Bessler M. Unintentional creation of reverse peristaltic alimentary limb during Roux-en-Y gastric bypass surgery. Surgery for Obesity and Related Diseases 2006;2:478–82. 16925386 10.1016/j.soard.2006.04.231

[8] Sherman V, Dan AG, Lord JM et al. Complications of gastric bypass: avoiding the Roux-en-O configuration. Obes Surg 2009;19:1190–4. 10.1007/s11695-009-9875-x 19506984

[9] Sanders CM, Neff M, Balsama L. Surgical treatment of retrograde peristalsis following laparoscopic Roux-en-Y gastric bypass. JSLS: Journal of the Society of Laparoendoscopic Surgeons 2012;16:469–72. 23318076 10.4293/108680812X13462882736574 PMC3535792

[10] Mitchell MT, Pizzitola VJ, Knuttinen MG et al. Atypical complications of gastric bypass surgery. Eur J Radiol 2005;53:366–73. 10.1016/j.ejrad.2004.12.016 15741010

[11] Mitchell MT, Gasparaitis AE, Alverdy J et al. Identification and management of an errant antiperistaltic Roux limb after total gastrectomy. J Gastrointest Surg 2006;10:622. 10.1016/j.gassur.2005.09.028 16627232

[12] Jedamzik J, Bichler C, Felsenreich DM et al. Conversion from one-anastomosis gastric bypass to Roux-en-Y gastric bypass: when and why—a single-center experience of all consecutive OAGB procedures. Surg Obes Relat Dis 2022;18:225–32. 10.1016/j.soard.2021.10.019 34794865

[13] Landreneau JP, Barajas-Gamboa JS, Strong AT et al. Conversion of one-anastomosis gastric bypass to Roux-en-Y gastric bypass: short-term results from a tertiary referral center. Surg Obes Relat Dis 2019;15:1896–902. 10.1016/j.soard.2019.09.059 31611182

[14] Carbajo MA, Luque-de-León E, Jiménez JM et al. Laparoscopic one-anastomosis gastric bypass: technique, results, and long-term follow-up in 1200 patients. Obes Surg 2017;27:1153–67. 10.1007/s11695-016-2428-1 27783366 PMC5403902

[15] Rutledge R . The mini-gastric bypass: experience with the first 1,274 cases. Obes Surg 2001;11:276–80. 10.1381/096089201321336584 11433900

